# Convergence of biomarkers and risk factor trait loci of coronary artery disease at 3p21.31 and HLA region

**DOI:** 10.1038/s41525-021-00174-z

**Published:** 2021-02-11

**Authors:** Majid Nikpay, Ruth McPherson

**Affiliations:** 1 Ruddy Canadian Cardiovascular Genetics Centre, University of Ottawa Heart Institute, Ottawa, ON Canada; 2 Atherogenomics Laboratory, University of Ottawa Heart Institute, Ottawa, ON Canada

**Keywords:** Cardiovascular diseases, Genetic markers

## Abstract

Here we seek to identify molecular biomarkers that mediate the effect of risk factors on coronary artery disease (CAD). We perform a SNP-based multiomics data analysis to find biomarkers (probes) causally associated with the risk of CAD within known genomic loci for its risk factors. We identify 78 biomarkers, the majority (64%) of which are methylation probes. We detect the convergence of several CNS and lifestyle trait loci and their biomarkers at the 3p21.31 and human leukocyte antigen (HLA) regions. The 3p21.31 locus was the most populated region in the convergence of biomarkers and risk factors. In this region, we noted as the *BSN* gene becomes methylated the level of stomatin (STOM) in blood increases and this contributes to higher risk of CAD. In the HLA locus, we identify several methylation biomarkers associated with various CAD risk factors. SNPs in the *CFB* gene display a *trans*-regulatory impact on the GRIA4 protein level. A methylation site upstream of the *APOE* gene is associated with a higher protein level of S100A13 which in turn leads to higher LDL-C and greater CAD risk. We find *UHRF1BP1* and *ILRUN* mediate the effect of obesity on CAD whereas methylation sites within *NOS3* and *CKM* mediate the effect of their associated-risk factors on CAD. This study provides further insight into the biology of CAD and identifies a list of biomarkers that mediate the impact of risk factors on CAD. A SNP-based initiative can unite data from various fields of omics into a single network of knowledge.

## Introduction

**Fig. 1:**
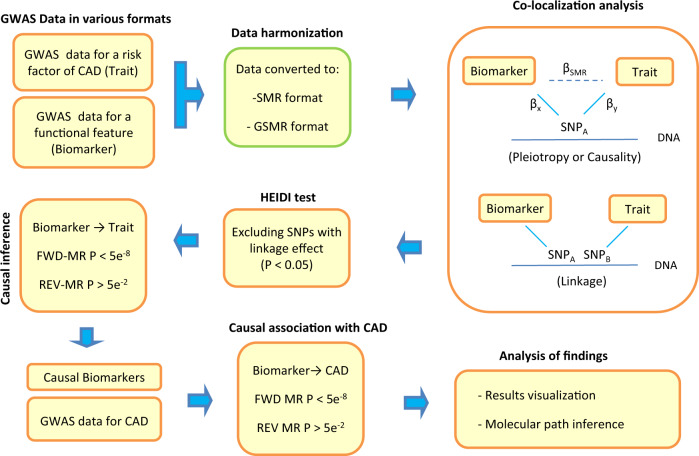
Flowchart of our SNP-based multiomics data analysis approach to identify biomarkers that mediate the effect of risk factors on CAD. Through this data analysis pipeline, we aimed to find biomarkers causally associated with the risk of CAD within known genomic regions for its risk factors. We started by collecting full GWAS summary statistics from studies that made their data publicly available. Next, we harmonized the data by converting them to SMR and GSMR formats. We then did the co-localization analysis to find SNPs that exert their effect through a molecular biomarker (probe). Next, we subjected the tagged probes to MR analysis to find biomarkers that are causally associated with a risk factor. Furthermore, we investigated whether the identified biomarkers are causally associated with the risk of CAD or other biomarkers for molecular insight.

GWAS studies have identified numerous loci associated with various complex phenotypes including the susceptibility to coronary artery disease (CAD). Multiomic approaches to the analysis of GWAS data provide a new means to understand the biology of these traits. Namely, we have GWAS data that catalog the associations between genome and the phenome but cannot provide molecular insight and we also have GWAS data from omics studies that report associations between genome and various molecular features. By combining these two different sets of data, it is possible to identify genomic regions where SNP-association signals are consistent (co-localize) for a trait and a molecular feature (biomarker), then Mendelian randomization (MR) can be used to test whether change in the level of the biomarker is causally contributing to the trait (Fig. 1 and Supplementary Fig. 1). A similar design can relate and combine different sets of omics data to understand the whole organism at molecular and cellular levels.

We have used GWAS data to investigate the genetic architecture of CAD and reported it largely derives from the cumulative effect of common SNPs throughout the genome each of small effect size^1,2^. We also reported that CAD is the outcome of several phenotypically distinct but genetically inter-related risk factors^3^. We devised a SNP-based analysis plan based on the above paradigm to identify biomarkers contributing to the risk of CAD^4,5^. In this study, we have extended these studies to identify genomic loci through which CAD risk factors exert their effects.

## Results

### Overview

Using our SNP-based multiomics data analysis plan (Fig. 1), we identified 78 biomarkers associated with various risk factors for CAD at GWAS significance level (*P* < 5e^−8^) as well as to CAD per se after correction for multiple testing (Supplementary Data 3). Although the mQTL datasets had the smallest sample sizes (Supplementary Data 1), majority of the identified biomarkers were methylation probes (68%). Transcription and protein probes accounted for 26% and 6% of the biomarkers, respectively. Summary association statistics for SNPs that we used to carry out these tests are available in Supplementary Data 4 and Supplementary Data 5.

We then obtained data from additional omic studies that also made their GWAS data publicly available and used this information to examine our results. The list of biomarkers that showed significant association (*P* < 0.05) following MR analysis is available in Supplementary Data 6. Of note, a lack of replication for a biomarker does not indicate a negative result. This is mainly because we used highly stringent statistical criteria to do the MR analysis. Namely, SNPs that (a) are in linkage equilibrium with each other (*r*^2^ < 0.05); (b) associated with the biomarker at *P* < 5e^−8^; and (c) are not showing pleiotropic effect (HEIDI *P* < 0.01). Therefore, for number of biomarkers, we did not obtain enough SNPs to do the MR analysis. For example, we did not replicate the association of SELE protein level with CAD using data from Suhre et al.^6^. However, after relaxing our LD threshold to r^2^ < 0.2, we found a higher level of SELE is causally contributing to lower risk of CAD [*B* = −0.05, *P* = 5e^−13^, Number of SNPs (*N*_SNPs_) = 6] using pQTLs from this study.

Our analysis plan can provide insight at both molecular (Biomarker → Biomarker) and clinical levels (Biomarker → Trait); therefore, findings from these studies can be used to investigate our results as well. For example, we find that SNPs near *SORT1* on chromosome 1 displays trans-regulatory impact on *GRN* on chromosome 17 (Supplementary Data 3) and this affects circulating levels of HDL. This finding is not unprecedented. SORT1 is an extracellular receptor for GRN^7^. In addition, it is known that GRN contributes to the anti-inflammatory effect of HDL by forming a complex with HDL/apolipoprotein A-I^8^.

Among our identified biomarkers, seventeen were associated with CAD at GWAS significance (Supplementary Fig. 2A). We also noted the presence of several *trans*-regulatory effects (Supplementary Fig. 2B). Studying these *trans*-regulatory effects is important because they can provide novel insights into the molecular pathway whereby a biomarker exerts its effect. Below we review the most notable findings:

### LDL and S100A13

We found the plasma protein level of S100A13 (S100 calcium-binding protein 13) is causally associated with higher LDL (*B* = 0.25, *P* = 4.75e^−55^) and higher risk for CAD (*B* = 0.05, *P* = 9.78e^−12^, Supplementary Data 3). The interpretation of β estimate from the MR analysis can be explained in standard deviation (SD) units. Therefore, a β = 0.05 (OR = 1.05) indicates that individuals whose S100A13 plasma protein levels are 1 SD above the population mean will have 1.05 times increase in risk to CAD.

**Fig. 2:**
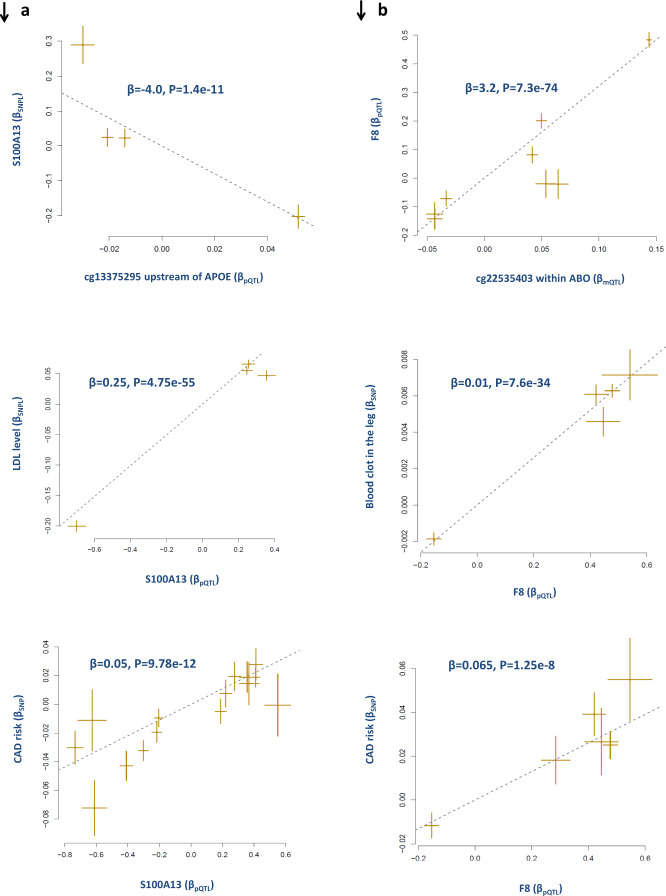
Molecular path whereby S100A13 and F8 impact CAD. **a** We found lower methylation at cg13375295 site, upstream of APOE, increases the level of S100A13 in the blood; this consequently contributes to higher level of LDL and higher risk of CAD. **b** MR analysis also revealed higher metylation of ABO locus (see Supplementary Data 8 as well) is associated with higher level of F8 and this increases the risk of CAD by increasing the risk of thrombosis. Each point represents a SNP, the *x*-value of a SNP is its Beta effect size on a molecular probe and the horizontal error bar, represents the standard error around the Beta. The *y*-value of the SNP is its Beta effect size on a biomarker/trait and the vertical error bar represents the standard error around its Beta. The dashed line represents the line of best fit (a line with the intercept of 0 and the slope of β from the MR test).

We noted that the S100A13 protein level in the blood is under the trans-regulatory impact of SNPs at the *APOE* locus. Further analysis revealed, both *APOE* and *S100A13* levels are under the regulatory impact of a methylation site (cg13375295) upstream of the *APOE* gene (Fig. 2). Higher methylation at this site was associated with lower levels of APOE (β = −3.7, *P* = 9.7e^−10^) and S100A13 (β = −4.0, *P* = 1.4e^−11^) in the blood. Previous studies also reported the *trans*-regulatory effect of APOE on S100A13^9,10^. MR analysis revealed that this gene has a causal impact on other cardiometabolic risk factors of CAD. We found that a higher level of S100A13 in the blood is associated (*P* < 5e^−8^) with higher levels of TC, immature reticulocyte fraction, and a higher risk of T2D (Supplementary Fig. 2 and Supplementary Data 7). The protein encoded by this gene is a member of the S100 family of proteins. It exhibits calcium and lipid-binding properties and is highly expressed in the heart. In smooth muscle cells, S100A13 co-expresses with other family members in the nucleus and in stress fibers, suggesting diverse functions in signal transduction.

### Thrombosis and F8

SNPs in the *ABO* locus are reported to have a *trans*-regulatory effect on protein levels of F8 (coagulation Factor VIII) in the blood. Factor VIII participates in the intrinsic pathway of blood coagulation; it is a cofactor for factor IXa which, in the presence of Ca^2+^ and phospholipids, converts factor X to the activated form Xa.

Here, we confirmed this effect (Supplementary Data 3) and noted that it is attributed to methylation sites within the *ABO* locus (Supplementary Data 8). We found multiple methylation sites within the *ABO* locus that show causal association with F8 protein levels. As displayed in Fig. 2, as *ABO* becomes methylated the level of F8 in the blood raises and this consequently increases the risk of thrombosis (*B* = 0.01, *P* = 7.57e^−34^) and the risk for CAD (*B* = 0.06, *P* = 1.25e^−08^).

### Obesity and UHRF1BP1

**Fig. 3:**
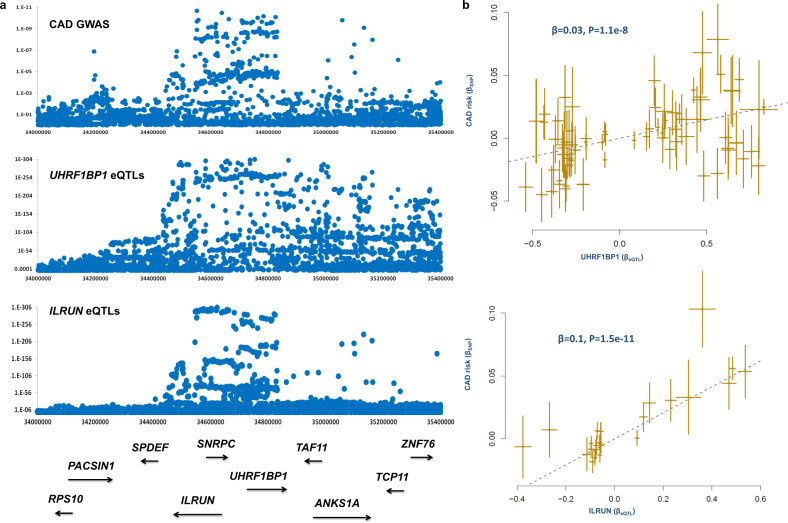
Higher expression of UHRF1BP1 and ILRUN are associated with higher risk of CAD. **a** Plotting GWAS data for CAD, and eQTLs for UHRF1BP1 and ILRUN revealed similar patterns. **b** MR analysis showed in this region subjects that are genetically susceptible to have higher expression of UHRF1BP1 and ILRUN tend to have higher risk of CAD. Each point represents a SNP, the *x*-value of a SNP is its Beta effect size on a molecular probe and the horizontal error bar, represents the standard error around the Beta. The *y*-value of the SNP is its Beta effect size on CAD risk and the vertical error bar represents the standard error around its Beta. The dashed line represents the line of best fit (a line with the intercept of 0 and the slope of β from the MR test).

Co-localization and subsequent MR analysis found *UHRF1BP1* on chromosome 6 as a gene for which elevated expression (*B* = −0.013, *P* = 5.14e^−16^, Supplementary Data 3) contributes to the risk of obesity (defined here as lower whole body impedance). MR analysis revealed that elevated expression of *UHRF1BP1* is positively associated (*P* < 5e^−8^) with time spent watching TV but negatively (*P* < 5e^−8^) with phenotypes that lower the risk of CAD including birth weight, age at first child birth, education qualifications, and HDL levels (Supplementary Data 7). In this region, we note that GWAS data for CAD, eQTLs for *UHRF1BP1* and eQTLs for *ILRUN* show correlated patterns (Fig. 3). Furthermore, MR analysis confirmed that change in the expression of these genes causally contributes to the risk of CAD (Fig. 3). *UHRF1BP1* is binding protein 1 for *UHRF1* which regulates chromatin structure and gene expression. *ILRUN* (inflammation and lipid regulator with UBA-like and NBR1-like domains) has immune functions. It is a negative regulator of innate antiviral response, and acts as an inhibitor of proinflammatory and antimicrobial cytokines^11^.

Further interrogation of our findings revealed the presence of two hotspot genomic regions whereby SNPs underlying several biomarkers and risk factors of CAD show overlapping association patterns.

### 3p21.31 region

**Fig. 4:**
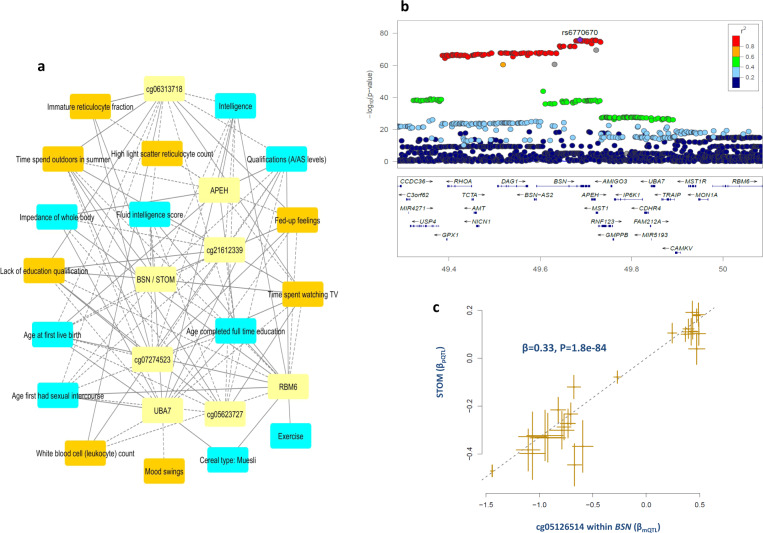
Convergence of molecular biomarkers and risk factors of CAD at chromosome 3p21.31 region. **a** Biomarker-risk factor associations, only biomarker-risk factor pairs with association *P*-value < 5e−8 (based on MR analysis) are displayed for better visualization. Risk factors that increase the risk of CAD are colored in orange, those that decrease the risk of CAD are colored in cyan and biomarkers (probes) are shown in yellow. The dashed lines indicate negative associations between probes and traits whereas solid lines indicate positive associations. Complete summary statistics are provided in Supplementary Data 7. **b** Regional association plot of SNPs that are associated with plasma protein level of STOM. SNPs are colored based on their linkage disequilibrium (LD) with the labeled top SNP (rs6770670), which has the smallest *P*-value in the region. LD (r^2^) calculations were based on the European population from the 1000 Genomes reference panel (Phase I; release 3). Genomic coordinates refer to the hg19 sequence assembly. **c** Higher methylation at BSN gene (measured by cg05126514) is causally associated with higher level of stomatin in the blood. Each point represents a SNP, the *x*-value of a SNP is its Beta effect size on cg05126514 and the horizontal error bar, represents the standard error around the Beta. The *y*-value of the SNP is its Beta effect size on stomatin level and the vertical error bar represents the standard error around its Beta. The dashed line represents the line of best fit (a line with the intercept of 0 and the slope of β from the MR test).

Our analysis pipeline (co-localization and subsequent MR analysis) revealed several methylation and expression biomarkers for CNS and lifestyle risk factors for CAD within the 3p21.31 region (Supplementary Data 3). This suggests that genes in this region may be involved in the neural process consistent with other evidence for *BSN, IP6K1, APEH, RBM5, and RBM6*. MR analysis revealed that the identified biomarkers are causally contributing to numerous risk factors of CAD (Supplementary Data 7). The 3p21.31 region was the most populated locus in terms of the convergence of molecular biomarkers and risk factors for CAD. Among the 78 identified biomarkers, those with the highest number of associated-risk factors were in this region (Supplementary Data 7). For example, expression biomarkers for *RBM6* and *UBA7* were associated with 16 CAD risk factors at GWAS significance level (Supplementary Data 7 and Fig. 4).

We noted that SNPs within *BSN* also display a *trans-*regulatory impact on the plasma protein level of stomatin (STOM). These data show that as this gene becomes methylated (as measured by cg05126514), the plasma protein level of STOM increases (*B* = 0.33, *P* = 1.83e^−84^, Fig. 4). We find that higher plasma levels of stomatin are positively associated with CAD and several CAD risk factors and negatively with CAD protective factors (Supplementary Data 7). Of note, higher methylation at cg05126514 site was also associated with higher risk of CAD (*B* = 0.06, *P* = 1.4e^−8^). Stomatin encodes a member of a highly conserved family of integral membrane proteins. The encoded protein localizes to the cell membrane of red blood cells and other cell types, where it may regulate ion channels and transporters. Loss of localization of the encoded protein is associated with hereditary stomatocytosis, a form of hemolytic anemia. Although the wide distribution of stomatin and its constitutive expression suggest an important role for this protein in cell biology, perhaps as a house-keeping component, its function remains to be clarified. In this study, stomatin was mainly associated with CNS and lifestyle risk factors (Supplementary Data 7) suggesting a CNS function for this gene.

Among the other identified biomarkers in this region, change in expression of *APEH* was associated with CAD at GWAS significance. The gene has aminoacylase activity and is implicated in various biological processes. Concordantly, MR analysis revealed higher expression of *APEH* to be negatively associated with the risk for obesity, immature reticulocyte count and positively with CNS/lifestyle traits associated with higher cognition (Supplementary Data 7).

### HLA region

Another congested region with regard to the convergence of risk factors for CAD and their biomarkers is the HLA region. Unlike the 3p21.31 locus, biomarkers identified in this region were associated to a more diverse group of risk factors (Supplementary Data 7). Following co-localization, and MR analysis, we identified 12 methylation biomarkers for white blood cell count, diabetes, obesity, height, and frequency of tiredness within the HLA region (Supplementary Data 7, Fig. 5).

**Fig. 5:**
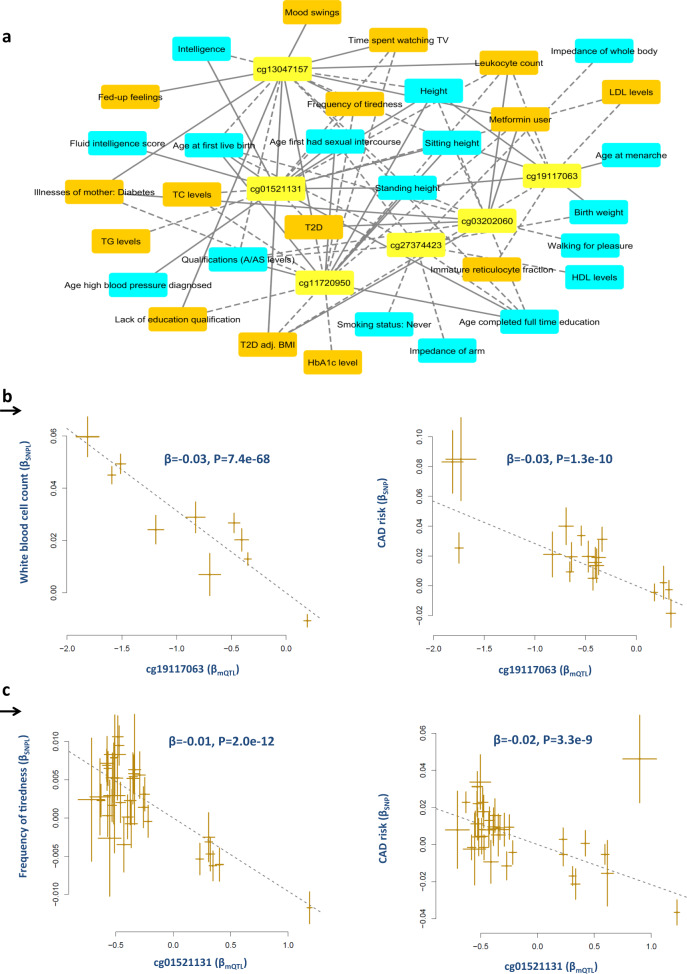
Convergence of methylation biomarkers and risk factors of CAD at HLA region. **a** Biomarker-risk factor associations, only half (N=6) of the biomarkers and biomarker-risk factor with association *P*-value < 5e−8 (based on MR analysis) are displayed for better visualization. Risk factors that increase the risk of CAD are colored in orange, those that decrease the risk of CAD are colored in cyan and biomarkers (probes) are shown in yellow. The dashed lines indicate negative associations between probes and traits whereas solid lines indicate positive associations. Complete summary statistics are provided in Supplementary Data 7. **b** Co-localization and MR analysis found cg19117063 to be associated with white blood cell count. We found higher methylation at this site is associated with lower leukocyte count and lower risk of CAD. **c** Similarly higher methylation at cg01521131 site was associated with lower frequency of tiredness and risk of CAD. Each point on the scatter plots represents a SNP, the *x*-value of a SNP is its Beta effect size on a molecular probe and the horizontal error bar, represents the standard error around the Beta. The *y*-value of the SNP is its Beta effect size on a molecular probe/CAD risk and the vertical error bar represents the standard error around its Beta. The dashed line represents the line of best fit (a line with the intercept of 0 and the slope of β from the MR test).

cg19117063 and cg11530659 associated with white blood cell count and cg01521131 associated with the frequency of tiredness were associated with CAD at GWAS significance (Fig. 5). In this region, we note SNPs within *CFB* (complement factor B) gene display trans-regulatory effect on synaptic gene *GRIA4*. MR analysis indicates that the level of this protein in blood shows the correlation with biomarkers within the HLA region (Supplementary Data 9). Furthermore, we note that this biomarker is causally associated with 12 CAD risk factors at GWAS significance. A higher level of this protein was positively associated with a higher risk for CAD, TC levels, white blood cell counts and negatively with height and CNS/lifestyle traits associated with higher cognition (Supplementary Data 7). GRIA4 encodes glutamate receptor 4 which is a member of a family of glutamate receptors that mediate fast synaptic excitatory neurotransmission. Glutamate receptors are the predominant excitatory neurotransmitter receptors in the mammalian brain and are activated in a variety of normal neurophysiologic processes. This finding also highlights the close molecular crosstalk between the immune and central nervous systems with regard to CAD risk factors.

## Discussion

CAD is a global health problem mandating improved strategies for risk assessment and prevention. Here we devised a SNP-based multiomics data analysis approach to identify biomarkers that are causally associated with risk for CAD within genomic regions that are known to be associated with its risk factors.

We identified 12 methylation biomarkers associated with various risk factors of CAD within the HLA region. This is the most important area in the genome regarding infection and autoimmunity and is essential in adaptive and innate immunity. Previously, we reported that SNPs that contribute to the risk of CAD are highly enriched in genomic regions pertinent to immune function^2^. In another study, we did a comprehensive phenome-wide search for risk factors of CAD. Although the most represented category was lifestyle features, CAD showed the highest genetic correlation with thrombotic conditions^3^. Taken together, these data support the notion that CAD involves an immune response to the cumulative effects of adverse lifestyle risk factors.

**Fig. 6:**
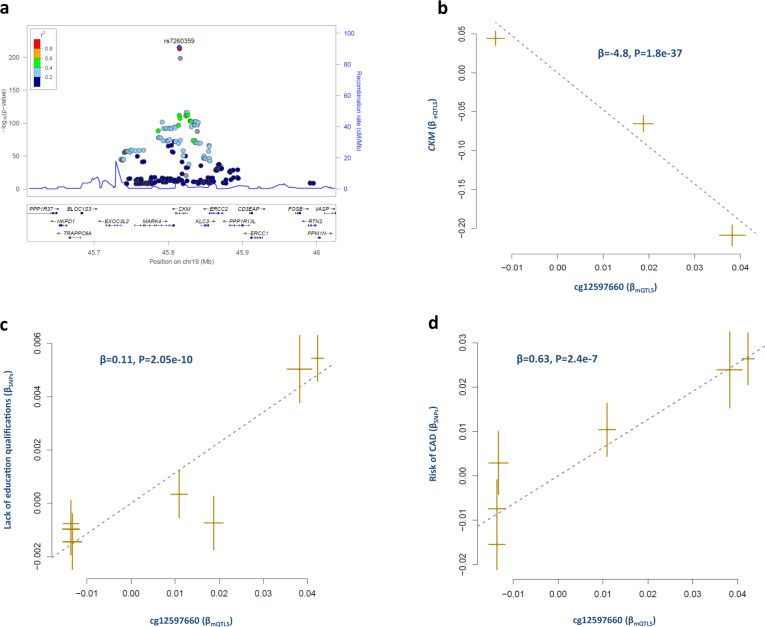
Methylation of CKM gene is associated with higher risk of CAD. **a** Regional association plot of mQTLs for cg12597660. SNPs are colored based on their linkage disequilibrium (*r*^2^) with the labeled top SNP (rs7260359), which has the smallest *P* value in the region. **b** We found higher methylation at cg12597660 (within CKM gene) is associated with lower expression of CKM. co-localization and MR analysis revealed cg12597660 mediates the impact of lack of education qualifications on CAD. **c** Higher methylation at this site was associated with lack of educational qualifications, and (**d**) higher risk of CAD. Each point represents a SNP, the *x*-value of a SNP is its Beta effect size on cg12597660 and the horizontal error bar, represents the standard error around the Beta. The *y*-value of the SNP is its Beta effect size on a biomarker/trait and the vertical error bar represents the standard error around its Beta. The dashed line represents the line of best fit (a line with the intercept of 0 and the slope of β from the MR test).

Within the HLA region, SNPs in the complement factor B gene (*CFB*) have a *trans*-regulatory impact on GRIA4 protein level in blood and this in turn is associated with the risk of CAD and its attendant risk factors (Supplementary Data 7). GRIA4 is a neural gene involved in synaptic transmission. This finding gives further support to studies that reported HLA contributes to neuronal function and development^12^. We also noted *STOM* a gene with hemo-immune function to be under the *trans*-regulatory effect of SNPs within the *BSN* gene that has a neural function. Although biomarkers within 3p21.31 co-localized with CNS/lifestyle trait loci, genes in this region appear to have diverse functions, for example, *APEH* is involved in barbiturate dependence, kidney cortex necrosis, gene expression, and innate immune response. Microdeletions in this region are characterized by developmental delay, elevated serum creatine kinase levels, and white matter involvement^13^. These findings may also explain why the biomarkers identified in this region were associated with multiple CAD risk factors (Supplementary Data 7). Furthermore, we noted that a number of seemingly non-CNS functional elements co-localize with CNS/lifestyle trait loci and mediate their impact on CAD. We found a methylation site within the HLA region that was a risk locus for the frequency of tiredness (Fig. 5 and Supplementary Data 3). A methylation site within Creatine Kinase, Muscle-Type (*CKM*) mediated the impact of lack of education qualifications (LEQ) on CAD. Higher methylation at this site was associated with a higher likelihood of LEQ, increased risk of CAD and lower expression of *CKM* (Fig. 6 and Supplementary Data 3). Altogether, these findings add support to the notion that CNS/lifestyle contributes to the risk of cardiovascular disease^14,15^.

Genomic regions that undergo epigenetic modification are considered as sites of gene-environment interactions. Previously we reported that SNPs that contribute to the risk of CAD are enriched in epigenetic sites associated with transcriptional activity^2^. Although in this study, the methylation datasets had the smallest sample size, majority 64% (*N* = 50) of the identified biomarkers were methylation probes. Biomarkers identified in the HLA region were all methylation probes. We also noted the impact of methylation on several known cardiometabolic biomarkers. In addition to those presented in the previous sections, we found a methylation biomarker (cg21901847, Supplementary Data 3) within *NOS3* that is associated with risk for early onset hypertension (β = −0.42, *P* = 5.3e^−10^) and CAD (β = 0.04, *P* = 7.5e^−5^). Similarly, a methylation site (cg18187658) within *GALNT2*, an established locus for TG and HDL-C^16^ was associated with a higher blood level of TG (β = 0.05, *P* = 1e^−19^) and the risk for CAD (β = 0.03, *P* = 8.6e^−6^). A methylation site within *ZNF664* was associated with higher HDL levels (β = 0.03, *P* = 9.4e^−11^), lower risk for obesity (β = −0.013, *P* = 3.5e^−16^) and CAD (β = −0.02, *P* = 2.5e^−5^). Recent studies also demonstrate that epigenetics may play an important role in the development of CAD^17–19^. Altogether, these findings indicate epigenetic reversal could have a profound impact in prevention and treatment of cardiovascular disease. In this regard, 3p21.31 and HLA region are prime candidates because they appear to be hotspots for interactions between risk factors of CAD and their biomarkers.

In this study, we connected different types of omics data (proteome, epigenome, transcriptome, phenome) to obtain novel molecular and clinical insights. We achieved this by linking these data to a single backbone i.e. genome or SNPs. This is a logical approach because DNA provides the information for the development and maintenance of various layers of omics and the human organism is the outcome of these layers and their connections. This study provides a new paradigm whereby the information generated in each field of biology can be linked to common SNPs. This can serve to unite the omics fields and provides a single network of knowledge to which the generated information is continually being linked.

Pursuant to our previous works^1–5^, this study provides further insight into the biology of CAD and identifies a list of biomarkers that mediate the impact of risk factors on CAD. The clinical message of these studies is that CAD (in its common form) is a complex disease arising from interactions amongst numerous genes and risk factors (notably lifestyle traits). Here we substantiate the hypothesis that CAD evolves in part from an immune response to the cumulative effects of adverse lifestyle factors on epigenetic modifications. We identify two hotspots (3p21 and HLA region) in the genome where there is a convergence between biomarkers and risk factor trait loci for CAD.

## Methods

### SNP-based approach toward linking omics data

Omics studies use probes (biomarkers) to study a phenomenon. The probes that are used in these fields are different, making it difficult to link the findings generated in each field. However, the common feature amongst these studies is that they measure genomic-related entities. Hence the genome is the connecting backbone. This approach is logical given that DNA provides information for the development and maintenance of various layers of omics and the living organism is the outcome of these layers and their connections.

Natural variations in the genome (SNPs) are historically used to study the genetics of omics and notably the phenome. Summary association statistics from these studies are usually publicly available. Therefore, if we can relate these findings through SNPs, we can repurpose them. For example, by linking the expression of a gene to the phenome and the epigenome, we can investigate the likely function of the gene and the location of regulatory elements that govern the expression of the gene. This allows us to understand the mechanism by which a gene impacts a phenotype. Our analysis pipeline (Fig. 1) was designed with the aim of combining omics data, in order to obtain such insights.

We started by asking the question, does the SNP-association signal for a risk factor and a functional element (Supplementary Data 1) co-localize in a genomic region. Supplementary Fig. 1 provides a graphical visualization for such a scenario. For this purpose, we systematically searched the genome and investigated the evidence of co-localization using the SMR (Summary-data-based Mendelian Randomization) test (version 1.03). The underlying assumption in this test is that, if in a region the effect of the top association signal (SNP_A_) on the risk factor is *β*_*y*_ and its effect on a biomarker is *β*_*x*_, we can estimate the effect of SNP_A_ on the risk factor that is attributed to the biomarker (*β*_SMR_) as^20^

1 $$\beta _{{\rm{SMR}}} = \frac{{\beta _y}}{{\beta _x}}$$

Then, we can test whether the computed effect significantly deviates from the null (SNP_A_ is not exerting its effect on risk factor through the biomarker) as^20^

2 $$T = \frac{{\beta _{{\rm{SMR}}}^2}}{{{\mathop{\rm{var}}} \left( {\beta _{{\rm{SMR}}}} \right)}} = \frac{{Z_y^2 \times Z_x^2}}{{Z_y^2 + Z_x^2}}$$

where *T*=${\upchi}_1^2$ and *Z*_*y*_ and *Z*_*x*_ are *Z*-statistics of SNP_A_ for the risk factor and the biomarker. The test is very useful because most published studies only provide access to summary level statistics and access to individual-level data is limited, due to privacy concerns and other logistical considerations.

From a biological perspective, a significant *β*_SMR_ could also be due to linkage or to a situation where the top signal, for the risk factor and the biomarker, are close but not the same (Fig. 1). To exclude such instances, we used Heterogeneity In Dependent Instruments (HEIDI) test as implemented in SMR software to exclude significant *β*_SMR_ effects that are due to linkage (*P*_HEIDI_ ≤ 0.05). The null model in the HEIDI test is that there is a shared casual variant that affects both the risk factor and the biomarker (pleiotropy or causality scenario) while the alternative hypothesis indicates linkage. The HEIDI test differentiates between these two scenarios by examining the change in pattern of $\frac{{\beta _y}}{{\beta _x}}$ for SNPs surrounding the SNP_A;_ a non-homogenous pattern indicates linkage.

Inherently, the SMR program cannot differentiate between pleiotropy (Biomarker ← SNP → risk factor) or causality (SNP → Biomarker → risk factor) because to make a causal inference between the exposure and outcome, multiple SNPs (reference points) are required. Therefore, we next performed multi-SNPs summary-based Mendelian randomization analysis, also known as 2-sample Mendelian randomization (where exposure and outcome are measured in two separate samples)^21^. This method requires summary association statistics and it works by contrasting the effect sizes of SNPs on the exposure with the effect sizes of the SNPs on the outcome. In this context, a significant positive association indicates subjects that are genetically susceptible to have higher values of the exposure (e.g. a biomarker) tend to have higher values of the outcome. SNPs that are used in the MR test must pass a number of criteria. Notably a) they must not be in linkage disequlibrium, b) must not show pleiotropic effect, and c) must be significantly associated with the exposure. For this purpose, we obtained summary association statistics (Beta and Standard error) for non-pleiotropic SNPs (*P*_HEIDI_ < 0.01) that are independently (*r*^2^ < 0.05) associated with a biomarker (*P* < 5e^−8^) and used these as an instrument to investigate their impact on the risk factor. To facilitate the process of SNP selection and the MR test, we used the GSMR (*Generalised Summary-data-based Mendelian Randomization*) algorithm implemented in GCTA software (version 1.92) and passing the default setting criteria as specified above^21^. As compared to other methods for 2-sample MR analysis, this algorithm automatically detects and removes SNPs that have a pleiotropic effect on both exposure and outcome; in addition, GSMR accounts for the sampling variance in β estimates and the linkage disequilibrium (LD) among SNPs, as such it is statistically more powerful than other 2-sample MR approaches^21^. Following this step (i.e. forward MR), we selected biomarkers causally contributing to risk factors (*P* < 5e^−8^) and subjected them to the reverse-MR (Risk factor → Biomarker). Namely, we identified independent SNPs that are associated with the risk factor (P < 5e^−8^) and contrasted their effect size on the risk factor with their effect size on the biomarker and excluded any risk factor-biomarker associations that showed significant evidence of reverse-causation (*P* < 0.05, Fig. 1). Biomarkers that also passed this step then were tested for their association with CAD by performing forward MR (Biomarker → CAD) and reverse MR (CAD → Biomarker). Biomarkers that passed this stage were then subjected to Bonferroni correction and those with forward MR (Bonferroni corrected *P* < 0.05) are reported in Supplementary Data 3.

### Data sources

Our analysis pipeline requires full GWAS summary statistics. As such, we searched for GWAS studies of molecular features (i.e. QTL studies) that made their full results publicly available (Supplementary Data 1). Next, we harmonized the retrieved data by converting them to SMR^20^ or GSMR^21^ format for downstream analyses. We obtained GWAS summary statistics for SNPs that influence the human blood proteome (pQTLs) from Sun et al.^22^ GWAS summary statistics for SNPs that influence transcriptome (eQTLs) were from Võsa et al.^23^ and GWAS summary statistics for SNPs that influence DNA methylation (mQTLs) were derived from two studies^19,24^. These studies are independent with reference to study participants and were conducted using blood samples. Although blood may not be the best tissue to study the biology of CAD, it appears to be an overall good proxy for many tissues. Liu et al.^25^ calculated pairwise genetic correlations (*r*_g_) of local gene expression among 10 different tissues from GTEX, and found the mean *r*_g_ between blood and other tissues is 0.71 (SD = 0.02).

We also obtained GWAS summary statistics for CAD from the most recent meta-analysis of CARDIoGRAMplusC4D and UK Biobank^26^, and GWAS data for the major CAD risk factors (Supplementary Data 2) from a recent phenome-wide study^3^. An important source of bias in genetic studies, including Mendelian randomization is population stratification. To address this issue, we collected GWAS summary statistics from studies carried out in European subjects and adjusted their results for the impact of population stratification. Our analysis pipeline also requires access to individual-level genotype data to estimate the linkage disequilibrium between SNPs. For this purpose, we used the genotype data from the INTERHEART study which is a sample of 854 subjects of European ancestry that we previously used in 1000 Genomes-based meta-analysis of CAD GWAS results^1^. The current study was conducted in accordance with the principles outlined in the Declaration of Helsinki. Research Ethics Board of the Ottawa Hospital approved the research protocols and all participants provided written informed consent.

### Reporting summary

Further information on research design is available in the Nature Research Reporting Summary linked to this article.

## Supplementary information

Supplementary Information

Supplementary Data 1

Supplementary Data 2

Supplementary Data 3

Supplementary Data 4

Supplementary Data 5

Supplementary Data 6

Supplementary Data 7

Supplementary Data 8

Supplementary Data 9

Reporting Summary

## Data Availability

Data that support the findings of this study are available from: https://github.com/mnikpay/Multiomics-MR-scripts.git.

## Code availability

Instructions and scripts to carry out the analyses are available from: https://github.com/mnikpay/Multiomics-MR-scripts.git.

## References

[1] Nikpay M (2015). A comprehensive 1000 Genomes-based genome-wide association meta-analysis of coronary artery disease. Nat. Genet..

[2] Nikpay M, Stewart A, McPherson R (2017). Partitioning the heritability of Coronary Artery Disease highlights the importance of immune-mediated processes and epigenetic sites associated with transcriptional activity. Cardiovascular Res..

[3] Nikpay M, Mohammadzadeh S (2020). Phenome-wide screening for traits causally associated with the risk of coronary artery disease. J. Hum. Genet..

[4] Nikpay, M. et al. Genome-wide identification of circulating-miRNA expression quantitative trait loci reveals the role of several miRNAs in the regulation of cardiometabolic phenotypes. *Cardiovascular Res.*10.1093/cvr/cvz030 (2019). 10.1093/cvr/cvz030 30715214

[5] Nikpay, M., Soubeyrand, S., Tahmasbi, R. & McPherson, R. Multiomics screening identifies molecular biomarkers causally associated with the risk of coronary artery disease. *Circulation: Genomic and Precision Medicine* **0**, 10.1161/CIRCGEN.119.002876 (2020). 10.1161/CIRCGEN.119.002876 32969717

[6] Suhre K (2017). Connecting genetic risk to disease end points through the human blood plasma proteome. Nat. Commun..

[7] Hu F (2010). Sortilin-mediated endocytosis determines levels of the frontotemporal dementia protein, progranulin. Neuron.

[8] Okura, H. et al. HDL/apolipoprotein AI binds to macrophage-derived progranulin and suppresses its conversion into proinflammatory granulins. *J. Atheroscler. Thromb.* **17**, 568–577 (2010). 10.5551/jat.3921 20215705

[9] Sebastiani, P. et al. A serum protein signature of APOE genotypes in centenarians. *Aging cell* **18**, e13023 (2019). 10.1111/acel.13023 PMC6826130 31385390

[10] Emilsson V (2018). Co-regulatory networks of human serum proteins link genetics to disease. Science.

[11] Ambrose RL (2020). Molecular characterisation of ILRUN, a novel inhibitor of proinflammatory and antimicrobial cytokines. Heliyon.

[12] Cebrián C, Loike JD, Sulzer D (2014). Neuronal MHC-I expression and its implications in synaptic function, axonal regeneration and Parkinson’s and other brain diseases. Front. Neuroanat..

[13] Eto K (2013). Microdeletions of 3p21. 31 characterized by developmental delay, distinctive features, elevated serum creatine kinase levels, and white matter involvement. Am. J. Med. Genet. A.

[14] Nikpay M, Turner AW, McPherson R (2018). Partitioning the pleiotropy between coronary artery disease and body mass index reveals the importance of low frequency variants and central nervous system–specific functional elements. Circulation: Genom. Precis. Med..

[15] Nikpay M (2012). Genetic mapping of habitual substance use, obesity-related traits, responses to mental and physical stress, and heart rate and blood pressure measurements reveals shared genes that are overrepresented in the neural synapse. Hypertension Res..

[16] Khetarpal SA (2016). Loss of function of GALNT2 lowers high-density lipoproteins in humans, nonhuman primates, and rodents. Cell Metab..

[17] Rizzacasa, B., Amati, F., Romeo, F., Novelli, G. & Mehta, J. L. Epigenetic Modification in Coronary Atherosclerosis. *J. Am. Coll. Cardiol.* **74**, 1352–1365 (2019). 10.1016/j.jacc.2019.07.043 31488273

[18] Steenaard RV (2015). Tobacco smoking is associated with methylation of genes related to coronary artery disease. Clin. epigenetics.

[19] McRae AF (2018). Identification of 55,000 replicated DNA methylation QTL. Sci. Rep..

[20] Zhu Z (2016). Integration of summary data from GWAS and eQTL studies predicts complex trait gene targets. Nat. Genet..

[21] Zhu Z (2018). Causal associations between risk factors and common diseases inferred from GWAS summary data. Nat. Commun..

[22] Sun BB (2018). Genomic atlas of the human plasma proteome. Nature.

[23] Võsa, U. et al. Unraveling the polygenic architecture of complex traits using blood eQTL metaanalysis. *bioRxiv* 447367, 10.1101/447367 (2018).

[24] Hannon E (2018). Leveraging DNA-methylation quantitative-trait loci to characterize the relationship between methylomic variation, gene expression, and complex traits. Am. J. Hum. Genet..

[25] Liu X (2017). Functional architectures of local and distal regulation of gene expression in multiple human tissues. Am. J. Hum. Genet..

[26] van der Harst P, Verweij N (2018). Identification of 64 novel genetic loci provides an expanded view on the genetic architecture of coronary artery disease. Circulation Res..

