# Supplementary material for: Convergence of biomarkers and risk factor trait loci of coronary artery disease at 3p21.31 and HLA region
Source: NPJ Genom Med. 2021 Feb 11;6:12. doi: 10.1038/s41525-021-00174-z (PMC7878768; doi:10.1038/s41525-021-00174-z)
Supplement: Supplementary file 1 — Supplementary Information [file 41525_2021_174_MOESM1_ESM.pdf]

Convergence of biomarkers and risk factor trait loci of coronary artery disease at 3p21.31 and HLA region

Majid Nikpay , Ruth McPherson

Supplementary information

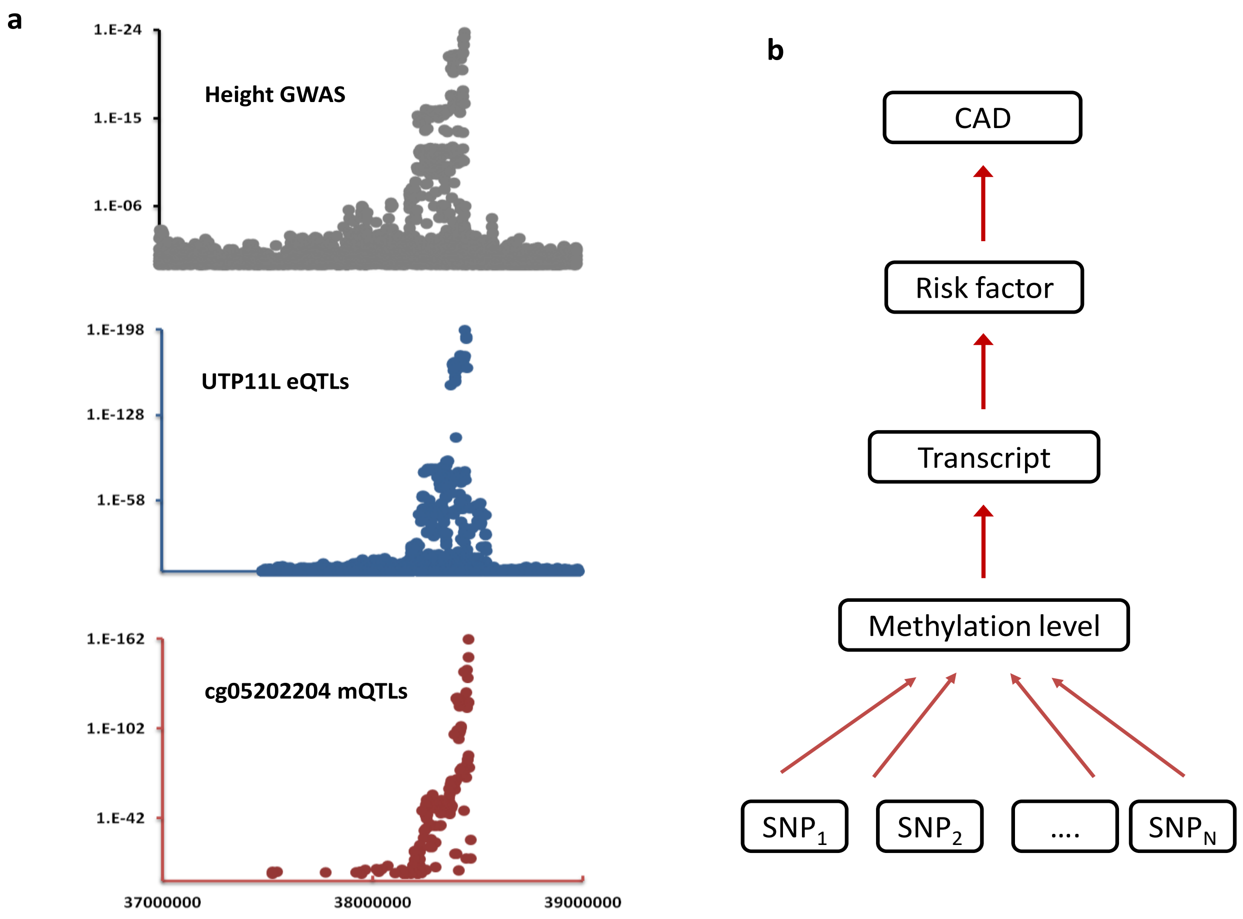

Supplementary Figure 1. Graphical representation of the concept of co-localization and MR within a genomic region. On chromosome 1p34.3 we found regional association plots for height, methylation and expression probes show similar patterns. a) co-localization analysis allows us to systematically identify such regions b) MR analysis then allows us to test for causality by leveraging multiple independent SNPs from this region, in order to obtain functional insight. Namely, we found in this region people that are genetically susceptible to have higher methylation at cg05202204 site tend to have lower expression of UTP11L, lower height and higher risk of CAD. Summary statistics are available in **Supplementary Data 3**. The Y-axis of regional association plots is -$\log_{10}$ (p-value) and the X-axis is position (bp).

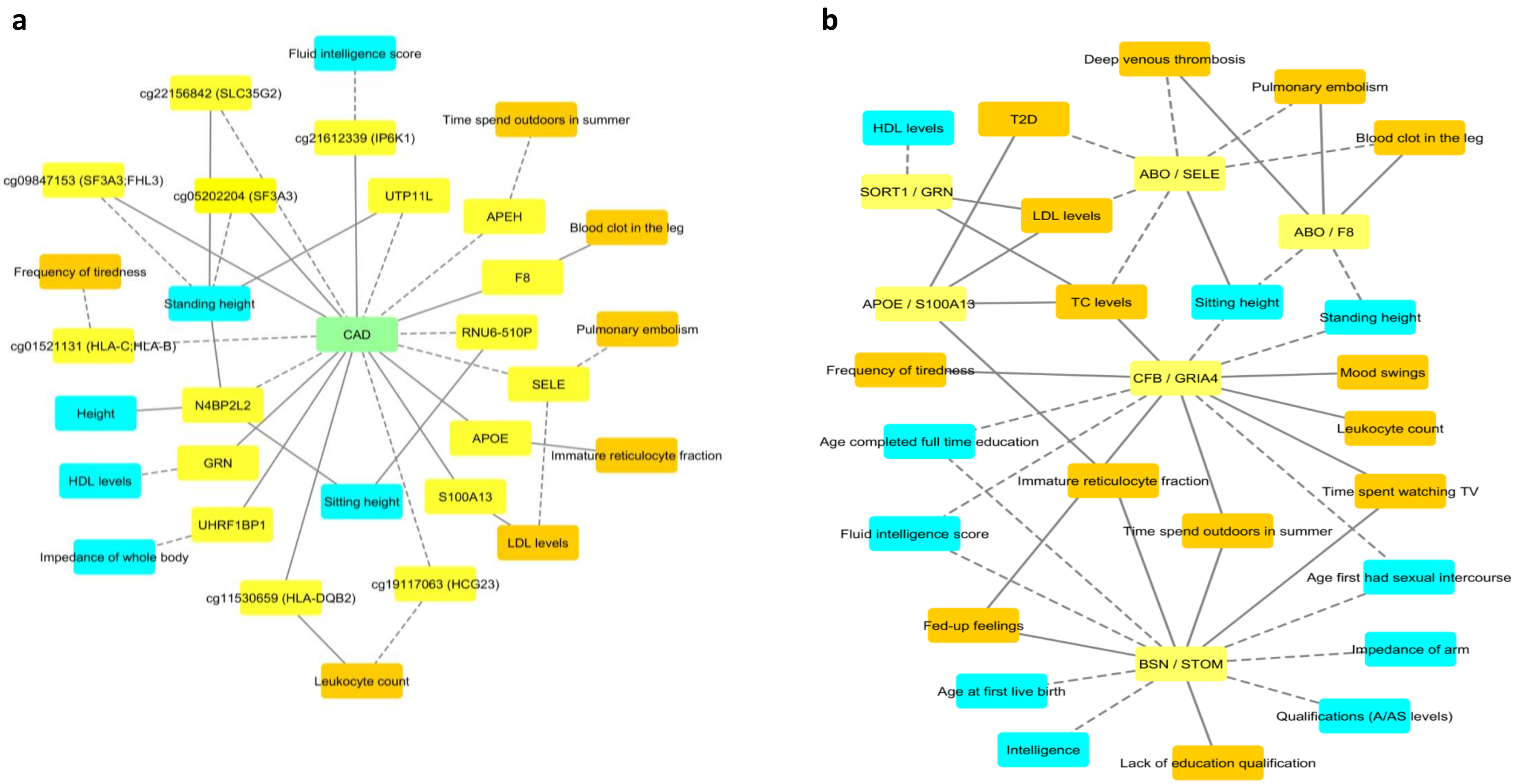

Supplementary Figure 2. Notable biomarkers that were associated with risk factors of CAD as wells as the risk of CAD. a) We found 17 biomarkers that were associated with CAD at GWAS significance level. b) Biomarkers that were under the trans-regulatory impact of other loci. Risk factors that increase the risk of CAD are colored in orange and those that decrease the risk of CAD are colored in cyan. Probes are shown in yellow and the genes assigned to methylation probes are written in parenthesis. The dashed lines indicate negative associations between probes and traits whereas solid lines indicate positive associations. Complete summary statistics are provided in **Supplementary Data 2 and 4**.

Supplementary Data

Supplementary Data 1. General characteristics of omics studies that we utilized in our analysis

Supplementary Data 2. List of traits that have causal effect on coronary artery disease (CAD) from PMID: 31907388

Supplementary Data 3. Summary association statistics for molecular biomarkers that are causally associated with risk factors as well as risk of CAD

Supplementary Data 4. Summary association statistics for SNPs that are used as an instrument to examine the effect of a biomarker on a risk factor

Supplementary Data 5. Summary association statistics for SNPs that are used as an instrument to examine the effect of a biomarker on CAD

Supplementary Data 6. Replication of the identified biomarkers using data from other omics studies

Supplementary Data 7. MR associations ($P<5e^{-8}$) between the identified biomarkers and the risk factors of CAD

Supplementary Data 8. Methylation of the ABO gene influences the F8 protein level in the blood

Supplementary Data 9. MR analysis revealed the level of the GRIA4 in blood correlates with biomarkers from the HLA region
